# Which Plants Used in Ethnomedicine Are Characterized? Phylogenetic Patterns in Traditional Use Related to Research Effort

**DOI:** 10.3389/fpls.2018.00834

**Published:** 2018-06-20

**Authors:** Estevão N. F. Souza, Elizabeth M. Williamson, Julie A. Hawkins

**Affiliations:** ^1^School of Biological Sciences, University of Reading, Reading, United Kingdom; ^2^School of Pharmacy, University of Reading, Reading, United Kingdom

**Keywords:** ethnopharmacology, Brazil, bioprospecting, leguminosae, traditional use, ethnobotany

## Abstract

Plants are important resources in healthcare and for producing pharmaceutical drugs. Pharmacological and phytochemical characterization contributes to both the safe use of herbal medicines and the identification of leads for drug development. However, there is no recent assessment of the proportion of plants used in ethnomedicine that are characterized in this way. Further, although it is increasingly apparent that plants used in ethnomedicine belong to preferred phylogenetic lineages, it is not known how this relates to the focusing of research effort. Here we identify species and lineages rich in ethnomedicinal use and develop methods to describe how well they are known pharmacologically and/or phytochemically. We find 50% of plant species of the family Leguminosae used in ethnomedicine in Brazil, a geographical area where plants are an important part of healthcare, have been the focus of either phytochemical screening or testing for biological activity. Plant species which have more use reports are studied significantly more often (*p* < 0.05). Considering the taxonomic distribution of use, 70% of genera that include species with ethnomedicinal use have been studied, compared to 19% of genera with no reported use. Using a novel phylogenetic framework, we show that lineages with significantly greater numbers of ethnomedicinal species are phylogenetically over-dispersed within the family, highlighting the diversity of species used. “Hotnode clades” contain 16% of species but 46% of ethnomedicinally-used species. The ethnomedicinal species in hotnode clades have more use reports per species (*p* < 0.05), suggesting they are more frequently used. They are also more likely to be characterized pharmacologically and/or phytochemically. Research focus has followed traditional use by these measures, at least for these Brazilian plants, yet ethnomedicinal species yielding candidate drugs, raising public health concerns and more intensively studied lie outside of the hotnode clades.

## Introduction

Plants are the primary health care resource in many communities around the world (Bannerman et al., 1983). They also serve as resources for the development of biomedicines, and traditionally-used species have contributed significantly to the development of biomedical drugs (Newman et al., 2000; Fabricant and Farnsworth, 2001; Cragg and Newman, 2013). It has been argued that ethnomedicinal use can be used to focus bioprospecting, addressing the decline in leads (Nwaka and Hudson, 2006) by provisioning new chemical entities or combinations of metabolites for testing (Gurib-Fakim, 2006; Sucher, 2013; Skirycz et al., 2016). Whether this is true or not, investigation of plants used in ethnomedicine is needed because public health may be compromised by ongoing use of some plants raises concerns (Jäger, 2015). Given these twin drivers of research, it might be expected that traditionally used plants are known rather well. However, there is a lack of knowledge about how well plants in general have been characterized; there are no explicit studies to investigate whether plants of ethnomedicinal importance are well known, and no recent data are available. The most cited source regarding the deficit of knowledge of plants comes from Verpoorte (1998), cited in Verpoorte (2000) and Fabricant and Farnsworth (2001). Verpoorte (1998) estimated that 6% of all plant species were screened for biological activity, and 15% evaluated phytochemically, but did not consider whether species were used in ethnomedicine. His estimates were made by compiling a count of the number of species in the NAPRALERT database against an estimate of total number of species.

Humans make direct use of only a proportion of the plant species around them, and it has long been known that plants with medicinal use are usually found more frequently in some families than in others (Moerman, 1991; Moerman et al., 1999). Phylogenetic methods are emerging as useful tools to describe these patterns (Saslis-Lagoudakis et al., 2011, 2012; Ernst et al., 2016). The well-known association of phytochemistry with taxonomy (Gibbs, 1974) is also increasingly better understood in terms of phylogeny (Wink, 2013). If lineages rich in species with ethnomedicinal use are those that have metabolites of interest, phylogenetic methods predict bioprospecting potential (Saslis-Lagoudakis et al., 2011; Ernst et al., 2016). Direct tests of phylogenetic structuring of phytochemistry for groups of ethnomedicinal and bioprospecting interest show that some (e.g., Rønsted et al., 2008) but not all (e.g., Grimbs et al., 2017) reveal phylogenetic signal. Nevertheless, it seems probable that close relatives of species of interest might yield larger quantities, or interesting variants, of valuable compounds.

The Leguminosae is one of the largest plant families (Lewis et al., 2005). With many documented uses, the family is over-utilized for medicine in some regions (Korea and Ecuador) but under-utilized in others (North America) (Moerman et al., 1999). Close-related species share similar secondary metabolites that could be of interest (Wink, 2003) and phylogenetic structure in the distribution of secondary metabolites has been well documented for the family (Wink, 2013). In Brazil, the Leguminosae comprises c. 2,800 species in more than 200 genera (Flora of Brazil 2020 in Construction, 2016). The species diversity, widespread distribution, and many reported uses (Souza and Hawkins, 2017), and the availability of phylogenetic information (Legume Phylogeny Working Group [LPWG], 2013) have prompted us to select the family as a case study. We focus on Brazil since its vast biodiversity is a potential source of new medicines as well as a source of primary health care: it is estimated that 66% of the population has no access to commercial drugs (Mazzari and Prieto, 2014).

The objective of this study was to provide a contemporary estimate of the extent to which the plants used in ethnomedicine have been investigated phytochemically and/or pharmacologically and relate this to the ethnomedicinal importance of the plant species and their lineages. Ethnomedicinal importance was estimated from the frequency of use reports in our existing compilation of ethnomedicinal Leguminosae in Brazil (Souza and Hawkins, 2017; www.ewedb.com). To identify ethnomedicinally important lineages, we made a phylogenetic study of the medicinal uses of our target group, the Leguminosae. Until now, phylogenetic investigations of ethnomedicinal plants have considered global floras (Halse-Gramkow et al., 2016), whole floras (e.g., Saslis-Lagoudakis et al., 2012) or genera throughout their range (e.g., Saslis-Lagoudakis et al., 2011; Ernst et al., 2016). As well as being the first study focused at family level, this study is also the first to study the frequency of reported use per species, in order to elucidate the phylogenetic relationships of ethnomedicinally important plants. Ultimately, we present a set of measures that show how research effort on plants with ethnomedicinal use, and their relatives, is focused.

## Materials and Methods

### Ethnomedicinal Use of Legumes in Brazil

Published ethnomedicinal use data compiled for the Leguminosae of Brazil (Souza and Hawkins, 2017; www.ewedb.com) were used for this study. Publications citing ethnomedicinal uses of Leguminosae species in Brazil were identified using online citation indices. We also used direct searches of unindexed journals, including the following journals not indexed by PubMed: Acta Botanica Brasilica, Flovet, Revista Brasileira de Biociencias, Revista Brasileira de Farmacognosia, Revista Brasileira de Plantas Medicinais, and Rodriguesia. Herbarium data was extracted from the online list of herbarium and biological collections from Brazil, Species Link^1^. The file was exported to excel format and a search using the keywords “medicinal” and “uses” (in Portuguese) was conducted to select only the specimens with ethnomedicinal information. Generic and species names followed the Flora of Brazil (Brazilian Flora, 2020), and were corrected (standardized) using the Plantminer R script (Carvalho et al., 2010).

### Pharmacology of Species Used in Brazil

Pharmacological data associated with the Brazilian ethnomedicinal species, and for all genera, whether used or not, were sought online using online citation indices including “Web of Science” (WoS). Search terms included the species name and commonly used synonyms, together with “pharmacolog^∗^”. The number of results from WoS for each species was recorded, together with publications related to pharmacological studies. As for ethnomedicinal use, names were standardized to follow the Flora of Brazil (Brazilian Flora, 2020).

### Analysis

From our surveys, we compiled data describing the presence or absence of use reports (citation of ethnomedicinal use in the literature) at species and generic level, and the number of use reports at species level. We scored whether each species was the subject of pharmacological research, and record research effort for each species as the number of published studies that investigate each species. We performed Spearman’s Rank Correlation test at species level to determine whether species with more use reports were the focus of more pharmacological research effort.

To understand the distribution of ethnomedicinal species in the family, a phylogenetic tree for the Leguminosae in Brazil was reconstructed at genus level, with one species per genus being sampled. Sequences from the DNA marker MatK were compiled from the Tree of Legumes (Legume Phylogeny Working Group [LPWG], 2013) when present, and from publicly available sequences on Genbank. All accession numbers are detailed in Supplementary Table S1. In total, 203 genera were sampled (92.7% of the total flora). Sequences were aligned using MUSCLE alignment available in Geneious 8.0 and adjustments were made manually. Sequence data was analyzed under the maximum-likelihood (ML) criterion, under the GRTGAMMA model as implemented in RAxML 7.2.8 (Stamatakis, 2014). Unsampled genera and species were added manually as polytomies using the R package PHYTOOLS and the function add.species.to.genus to generate a species tree (Revell, 2012). To make the tree ultrametric, we used the function chronos from the package APE (Paradis et al., 2004). The phylogenetic hypothesis obtained was compared to the Tree of Legumes (Legume Phylogeny Working Group [LPWG], 2013).

**FIGURE 1:**
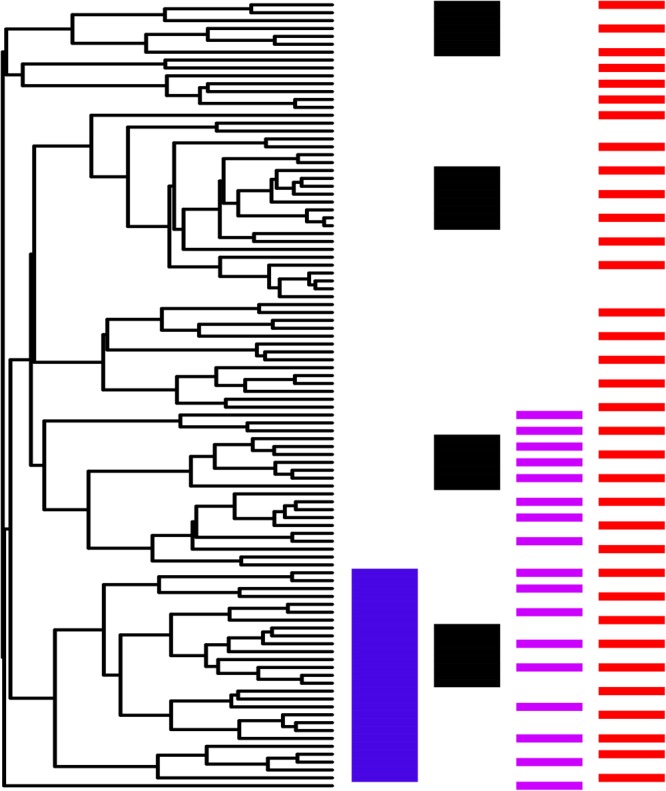
Schematic representation of scenarios of relatedness. Clustering and overdispersion are inferred from MPD and MNTD values (and their related NRI and NTI indices respectively). MPD describes relationships that occurred on deep nodes of the phylogeny, while MNDT reflects relationships at shallower nodes. Combined values can be used to infer different scenarios. Blue, phylogenetic clustering, MPD and MNTD positive; black, overdispersion of clustering, MPD negative and MNTD positive; pink, clustering of overdispersion, MPD positive and MNTD negative; red, phylogenetic overdispersion, MPD negative and MNTD negative. The phylogenetic tree was generated with the function “rtree” from the package ape using R.

Having confirmed our phylogeny was congruent with the existing hypothesis for the Leguminosae, the phylogenetic structure of ethnomedicinal uses was investigated using tools widely used in community phylogenetics (Pearse et al., 2014). We calculated the Net Relatedness Index (NRI) and Nearest Taxon Index (NTI) (Webb, 2000; Webb et al., 2002), with the functions ses.mpd and ses.mntd from the PICANTE package (Kembel et al., 2010). The chosen null model was “taxa.labels”, which shuffles the distance matrix labels across all taxa included in distance matrix with 999 runs. The NRI and NTI values were obtained by multiplying the MPD (Mean phylogenetic distance) and MNTD (Mean Nearest Taxon Distance) values by -1. The NRI value represents the average distance between a species and all other species with the same characteristic (in our case ethnomedicinal use). Higher values of NRI (negative for MPD) indicates a phylogenetic clustering of a sample, while negative values (positive for MPD) show an overdispersion of the studied taxa. Similarly, NTI (and MNTD) values can indicate clustering or dispersion (**Figure 1**). According to Webb (2000), NRI values describe a deep relationship in the phylogeny, which here might be evidence for clustering or overdispersal at tribal or sub familial levels. On the other hand, NTI describes relatedness in terminal clades, in our case clustering or overdispersion of congeneric ethnomedicinal plants (see also Mazel et al., 2016). Phylogenetic structure was investigated for the ethnomedicinal species compared to the whole Leguminosae flora in Brazil. “Hotnode clades” (as described by Saslis-Lagoudakis et al., 2012) were sought using the NODESIG function in also in R (R Core Team, 2015) adapted by Abellán et al. (2016).

The phylogenetic framework was used to address the following questions:

1. Are there more species that have been the focus of pharmacological study within the lineages that are hotnode clades for ethnomedicinal use?
2. Is intensity of use of species for ethnomedicine greater in the lineages that are in hotnode clades for ethnomedicinal use
3. Is the intensity of research effort greater within hotnode clades?
4. Do hotnode clades yield more drugs?

Intensity of ethnomedicinal use was estimated based on the number of citations/use reports in the database. Intensity of research effort was measured by counting the number of reported studies (publications) for each species. The intensity of research effort was compared for the species ethnomedicinally-used for medicine inside and outside of the hotnode clades. A Wilcoxon test was used to determine whether there are significantly more pharmacological studies outside of hotnode clades than would be expected if pharmacological effort was homogeneous.

To assess whether traditional use coincides with development of commercial drugs we extracted the list of genera yielding FDA-approved and clinical-trial drugs (drug genera) from the recent large-scale study of Zhu et al. (2011) and recorded the number of genera found in Brazil. We also recorded whether these genera were present in hotnode clades.

All underlying research data is available from the corresponding author.

## Results

### Data Description

**FIGURE 2:**
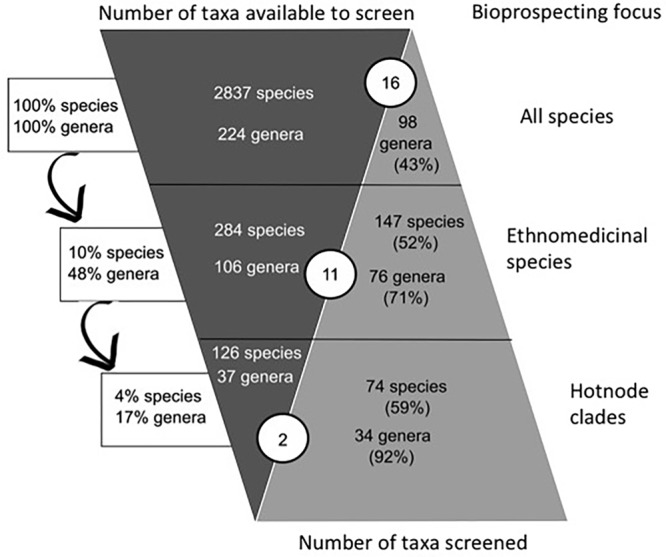
Comparison of ethnomedicinal selection and pharmacological research effort. The left “column” (dark gray) shows the decreasing numbers of potential leads, if focus is on taxa (middle row) or lineages (bottom row) with ethnomedicinal use. This column shows that a focus on ethnomedicinal hotnode clades reduces the size of the species pool from 2837 species in total to 126 species that have ethnomedicinal use and are inside a hotnode clade. The arrows show the proportions of total flora represented in the samples, for example, the 126 species with ethnomedicinal use in the bottom row represent 4% of all species. This is 4% of all species. The right “column” (light gray) shows the proportion of these taxa that have been screened according to our survey. If focus is on the whole flora, there are 98 genera that have been investigated pharmacologically (43% of all genera). Considering the ethnomedicinally important lineages (bottom row), there are 34 genera with ethnomedicinal use and have been characterized pharmacologically that belong to hotnode clades (92% of the genera with ethnomedicinal use and in hot modes). The circled numbers in blue show the number of species that have yielded drugs approved for use or clinical trial by the FDA. As focus on ethnomedicinally important groups increases, there is a concomitant increase in research effort, but fewer drugs approved for use or clinical trial by the FDA.

There were 286 species (10% of the total legume flora) in 104 genera (48% of the total legume genera) reported to have ethnomedicinal use (**Figure 2**). The pharmacological review identified 98 genera that had been screened (43% of genera). There were 143 ethnomedicinally-used species (50% of the species used) that had been the focus of research described in 3047 publications abstracted by the Web of Science. The ethnomedicinally-used species that had been screened are found in 77 genera, thus 72% of genera with ethnomedicinal use had been the focus of pharmacological study. A much lower percentage of the 118 genera without any ethnomedicinal use had been studied (22 genera, 19%). The Spearman’s Rank Correlation showed significant positive correlation between use and pharmacological study at the species level (*p* < 0.05; ρ = 0.36).

### Phylogenetic Analysis

**FIGURE 3:**
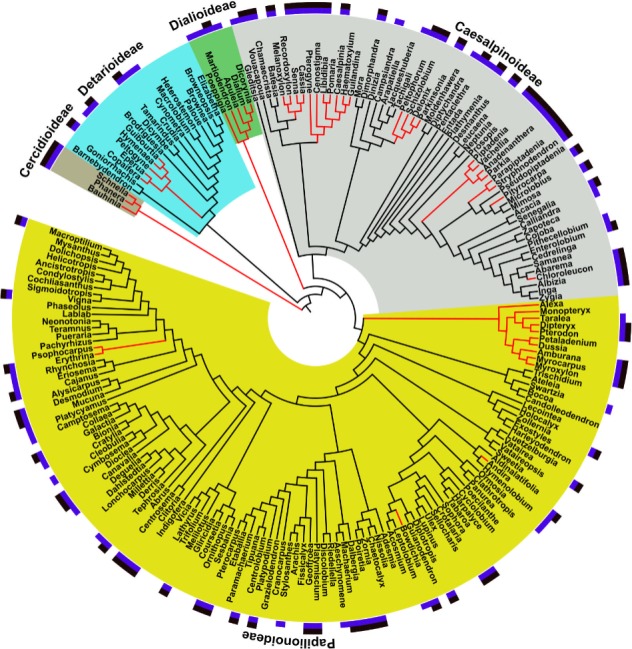
Distribution of medicinal uses and pharmacological studies in the Leguminosae phylogeny and “Hotnode clades” for ethnomedicinal use. Block colors at the tips: purple, ethnomedicinal use for that genus; black, pharmacological studies for that genus. Branch colors: red branches, genera present in hotnode clades for species with ethnomedicinal use. Clade colors: yellow, grey, green, light blue and brown indicate membership of subfamilies. Figure prepared using iTOL online (Letunic and Bork, 2016).

The phylogeny we reconstructed (Supplementary File S1) was congruent with current hypotheses of relationship for the Leguminosae. The investigation of the phylogenetic structure of ethnomedicinal species for medicine shows an over-dispersed structure for overall ethnomedicinal use (NRI and NTI < 0, *p* < 0.05) (**Figure 3**). The same pattern of overdispersal was found for therapeutic applications considered separately (Supplementary Table S2). The Nodesig analysis showed 126 ethnomedicinal species (44% of species with at least one use report) were inside hotnode clades, but only 16% of the total flora (**Figure 2** and **Table 1**).

**Table 1:** Characterization of “hotnode clades” in terms of absolute numbers and proportions of species and studies.

	Hotnode clades	Outside
Number of genera with ethnomedicinal use	37 (74%)	65 (42%)
Number of genera with pharmacological study	34 (68%)	60 (39%)
Number of species with ethnomedicinal use	126 (28%)	150 (6%)
Number of species with pharmacological study	74 (16%)	73 (3%)
Intensity of ethnomedicinal use (Number of citations)	1192	549
Intensity of pharmacological study (Number of studies)	1028	1665
Average number of studies per ethnomedicinal species	13	22

*Proportions are reported according to the total number of species in hotnode clades (446 species) and outside of hotnode clades (2331), and according to the total number of genera in hotnode clades (50) and outside hotnode clades (152). Genera with ethnomedicinal use are genera which include at least one species with one use report. Genera with pharmacological study include at least one species included in a publication describing pharmacological study. Citations refers to the number of use reports a species has in the database for ethnomedicinal use. Studies refers to the number of publications describing pharmacological study.*

**FIGURE 4:**
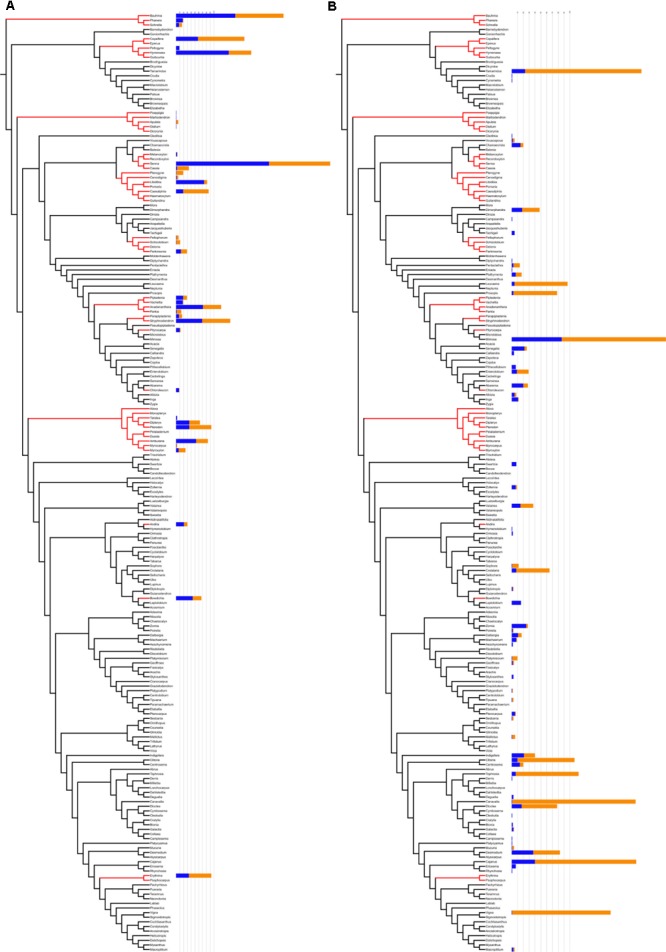
Comparison between hotnode clades **(A)** and other nodes **(B)** in relation to number of citations (blue) and number of pharmacological studies (orange). Hotnode clades are calculated based on the distribution of recorded use of species. Species in hotnode clades have significantly more use reports (*p* < 0.05). Figure prepared using iTOL online (Letunic and Bork, 2016).

1. There are more species that have been the focus of pharmacological study within the lineages that are hotnode clades for ethnomedicinal use. Of the 126 hotnode clade species with ethnomedicinal use, 74 (16% of all hotnode clade species) were the focus of at least one pharmacological study. A lower number, 73 species (3% of all non-hotnode clade species) of the 150 ethnomedicinal species outside hotnode clades had been screened (**Table 1**) revealing a preference for screening ethnomedicinal species that are in hotnode clades.
2. The intensity of ethnomedicinal use of species for medicine is greater in the lineages that are hotnode clades for ethnomedicinal use. Species with ethnomedicinal use present in the hotnode clades were cited almost twice as often as the ones outside the hotnode clades (*p* < 0.05; **Table 1**).
3. The intensity of research effort is not significantly greater for ethnomedicinal species inside hotnode clades. Although there were numerically more species from hotnodes screened, no significant difference was found for the intensity of screening between ethnomedicinal species inside and ethnomedicinal species outside of hotnode clades. In fact, ethnomedicinal species outside hotnode clades are more studied. **Figure 4** shows on a phylogeny how intensity of use and intensity of study are distributed.

4. Hotnode clades yield fewer drugs approved by FDA for use or clinical trials. We identified 16 genera found in Brazil that include species (not necessarily from Brazil) that have yielded drugs approved for use or clinical trial by the FDA. We found that 11/14 of these genera have species with ethnomedicinal use in Brazil, and that only 2/11 of these genera belong to hotnode clades. **Table 2** shows the therapeutic applications recorded for all Brazilian species belonging to these genera, and indicates whether each genus belong to a hotnode.

**Table 2:** The 16 genera found in Brazil that include species (not necessarily from Brazil) that have yielded drugs approved for use or clinical trial by the FDA and their ethnomedicinal use, if any, in Brazil.

Genera yielding drugs approved by FDA for use of clinical trial	Drug (therapeutic class or targeted disease) (Zhu et al., 2011)	Presence in hotnode?	Therapeutic application of species in that genus in Brazil (frequency of citation)
*Acacia*	Amphetamine (neurological disease)	No	DFS (1)
	Atenolol (cardiovascular disease)		
	Betaxololo HCI (cardiovascular disease)		
	Dextroamphetamin e sulfate (ADHD; narcolepsy)		
	Isoprenaline (Cardiovascular disease)		
	Methamphetamine (neurological disease)		
	Metoprolol tartrate (Cardiovascular disease)		
	Polyphenon 100 (Haemostatic)		
	Propranolol (Cardiovascular disease)		
	Tranylcypromine sulfate (Major depressive)		
	Vyvanse (Neurological disease)		
*Senegalia*	Atenolol (cardiovascular disease)	No	DFS (1), DMC (3), DRS (5), IPD(2)
	Betaxololo HCI (cardiovascular disease)		
	Amphetamine (neurological disease)		
	Dextroamphetamin e sulfate (ADHD; narcolepsy)		
	Isoprenaline (Cardiovascular disease)		
	Lisdexamfetamine dimesylate (ADHD)		
	Methamphetamine (neurological disease)		
	Metoprolol tartrate (Cardiovascular disease)		
	Propranolol (Cardiovascular disease)		
	Tranylcypromine sulfate (Major depressive)		
	Vyvanse (Neurological disease)		
*Cassia (Senna)*	Danthron (laxative) Sennoside A (Cardiovascular disease) Sennoside B (Cardiovascular disease)	Yes	Other (tanning) (Cassia). Senna— DBI (5), DCS (12), DDS (35), DFS (11), DGS (9), DMC (7), DNS (6), DRS (27), DSS (10), ENM (8), IPD (9), NEO (3), PCP (3)
*Melilotus*	Hidrosmin (Cardiovascular disease)	No	Medicinal
	Warfarin (Cardiovascular disease)		
*Mucuna*	L-Dopa (Neurological disease)	No	Medicinal
	Levodopa (dietary supplement)		
	Melevodopa (Neurological disease)		
*Crotalaria*	Monocrotaline (oncological disease)	No	DFS (1), IPD (1)
*Sophora*	Pachycarpine (Oxytocic)	No	Medicinal
	Sofalcone (Antiulcer)		
	Daidzein (oncological disease)		
	Phenoxodiol (Oncological disease)		
*Trifolium*	Pinitol (Expectorant)	No	not used as medicinal in Brazil
	Daidzein (oncological disease)		
	Phenoxodiol (Oncological disease)		
*Lonchocarpus*	Rotenone (Piscicide)	No	not used as medicinal in Brazil
*Phaseolus*	Daidzein (oncological disease)	No	ENM (1), IPD (1)
	Phenoxodiol (Oncological disease)		
*Pueraria*	Daidzein (oncological disease)	No	not used as medicinal in Brazil
	Phenoxodiol (Oncological disease)		
*Vigna*	Daidzein (oncological disease)	No	DSS (1)
	Phenoxodiol (Oncological disease)		
*Indigofera*	Indirubin (oncological disease)	No	DDS (2), DGS (2), DNS (1), PCP (1)
*Vachellia*	Pinitol (Expectorant)	Yes	DDS (2), DEA (1), DMC (2), DNS (1), DRS (2)

*For each genus we indicate the drug following Zhu et al., 2011, whether the genus is in a hotnode clade, the therapeutic applications of Brazilian species and the frequency of citation. Therapeutic applications are as follows: DBI, diseases of the blood and blood-forming organs and certain disorders involving the immune mechanism; DCS, diseases of the circulatory system, DDS, diseases of the digestive system; DFS, diseases of the femalegenito system; DGS, diseases of the genitourinary system; DMC, diseases of the musculoskeletal system and connective tissue; DNS, diseases of the nervous system; DRS, diseases of the respiratory system; DSS, diseases of the skin and subcutaneous tissue; ENM, endocrine, nutritional, and metabolic diseases; IPD, certain infectious and parasitic diseases; NEO, neoplasms; OTHER, not classified diseases such as fever, pain, and inflammation.*

## Discussion

This study reveals that half of the species of Leguminosae used in ethnomedicine in Brazil have been the focus of at least one phytochemical or pharmacological study. Despite the importance of ethnomedicinal plants in bioprospecting and local health care, the frequently cited global estimates of 9% of plants screened do not consider whether plants are used in ethnomedicine or not (Fabricant and Farnsworth, 2001). Although ethnomedicinal use is not taken into account, and this figure is now more than 15 years old, it probably remains our best estimate of how well known are the properties of ethnomedicinally important plants. Our study is an original insight into this question, and shows that at least for some ethnomedicinally important plant lineages in some parts of the world, there is now considerable scientific knowledge of the properties of plants. The consideration of the relationship between pharmacological study and intensity of ethnomedicinal use we present here is a timely first step towards a data-driven evaluation of how ethnomedicine might inform bioprospecting, or direct research into the health risks associated with local plant-based healthcare.

A wider audit is needed to determine whether our estimate of 50% of plants screened applies beyond the geographical and taxonomic limits of our study. Investment in natural products research informed by traditional knowledge has fluctuated (Koehn and Carter, 2005), and while there may be many factors at play, Brazil has invested in realizing the value of local and indigenous knowledge of indigenous plant resources through pharmacological research (Dutra et al., 2016). This might suggest that Brazilian plants could be rather well known compared to plants in other biodiversity hotspots. However, our findings should be considered in the light of Brazilian government policy to integrate herbal medicines into their healthcare system. Because of the huge number of plant species used medicinally in Brazil, the Health Ministry policy focused efforts on elucidating the efficacy and safety of 71 promoted ethnomedicinal plant species (Ministério da Saude, 2009; SUS, 2009; Mazzari and Prieto, 2014). It might be expected that there have been fewer resources directed towards characterization of non-list species, though seven of the listed species belong to family Leguminosae. That many species are found outside of Brazil and we did not limit our survey of research effort to Brazilian research groups might increase likelihood of screening of plants. The family Leguminosae is a rather well-known plant family taxonomically, so it will be interesting to see whether it is better-known than other families. Our study might serve as a start point from which an evolutionarily-informed audit of scientific knowledge of ethnomedicinal plants could be developed.

The approach taken here goes far beyond counting the number of species used in ethnomedicine and recording whether these plants are the focus of pharmacological studies. We use a phylogenetic framework to identify evolutionary lineages that are particularly important in ethnomedicine, and record whether species in those lineages are more often screened than other ethnomedicinal plants. A first step was the phylogenetic characterization of ethnomedicinal species of Leguminosae in Brazil plants, and we revealed novel patterns with significant implications for bioprospecting. Previous phylogenetic studies reported clustering of species with ethnomedicinal use, both for whole floras (Saslis-Lagoudakis et al., 2012), and when species level phylogenies are used to interrogate the patterns of plant use in genera (Saslis-Lagoudakis et al., 2011; Ernst et al., 2016). Our study is the first focused at the level of a plant family, and also the first to recover a signal of phylogenetic overdispersal (**Table 1**). Phylogenetically-informed bioprospecting until now has been concerned with clustering, whether recovered using D-statistics (Rønsted et al., 2008), Pagel’s lambda (Cámara-Leret et al., 2017), or community phylogenetic methods (Saslis-Lagoudakis et al., 2011, 2012, 2014; Yessoufou et al., 2015; Ernst et al., 2016; Halse-Gramkow et al., 2016). Overdispersal challenges the established use of phylogeny as a predictive tool to inform ethnopharmacological screening, but here we show that hotnode clades can still be identified and lineages of interest highlighted (**Figure 2**). Bioactivity of plant species is related to the presence of secondary metabolites that mediate interactions of plant species with pathogens and herbivores, other plants, and pollinators (Bourgaud et al., 2001; Edris, 2007; Heil, 2008; Huang et al., 2012). While some metabolites in the legumes, such as flavonoids, triterpenes, and pinitol, are of broad occurrence and can be found in diverse groups, others are limited in their phylogenetic distribution (Wink, 2013). The pattern of overdispersal we find here might result from the adoption of multiple lineages with different secondary metabolites for ethnomedicine. The interplay of chemical and phylogenetic diversity underscores that the hierarchical level of the study is important in devising and interpreting any study. Saslis-Lagoudakis et al. (2012) indicated in a study across all Angiosperms that plants of the Leguminosae were included in cross-cultural hotnode clades. However, in this study at the family level, overdispersal reveals phylogenetically non-random use, comprising overdispersed lineages that we hypothesis here are selected for their diverse chemistries. Many more studies are needed to determine how general these emerging patterns are, and whether they apply to less species-rich families. In the context of what might be overdispersed clusters (**Figure 1**) a fresh view of priorities for pharmacological research effort can be made.

Our data show how pharmacological studies are distributed relative to hotnode clades. Although hotnode clades comprise only 14% of total species they include almost half of the ethnomedicinal species. As well as being evolutionary hotspots for ethnomedicinal species, overall the hotnode clades encompass significantly more use reports. These same hotnode clades also include more than half of the ethnomedicinally used species that have been the focus of pharmacological investigation. Our study corroborates the view that ethnomedicinal use guides pharmacological research (Gurib-Fakim, 2006), showing that close relatives of ethnomedicinal plants are also the focus of study. Using a phylogenetic framework we highlight here how just a small part of the phylogeny concentrates both traditional knowledge and pharmacological research, whilst overall the diversity of ethnomedicinal species outside of hotnode clades are relatively poorly known.

Emphasis on few lineages would benefit bioprospecting if valuable leads are more likely to be encountered in hotnode clades. A total of 35 species belonging to 25 genera in family Leguminosae, 14 of which are found in Brazil, yield 44 of the products approved for clinical trial by the FDA (Zhu et al., 2011). Only two genera of the 15, *Senna* and *Vachellia*, fall into a Brazilian hotnode clade. This is in complete contrast to the findings of Saslis-Lagoudakis et al. (2012), who found a significant association between approval and membership of hotnode clades. The discrepancy might be attributed to the different hierarchical level of study, or to the choice of Leguminosae as a study group. Whilst the whole Leguminosae falls into a hotnode clade in the study of three floras (Saslis-Lagoudakis et al., 2012), focusing the study on the family means that lineages within the Leguminosae are discriminated. We suggest here that our hotnode clades include plants of mild action, whilst clinical investigation has prioritized species of relatively high toxicity. It is known, for example, that species of family Leguminosae used as contraceptives or abortifacients show high toxicity and yield anticancer therapeutics (Leonti et al., 2017). To illustrate the relationship between mild action and frequent use, we can contrast uses of species of two genera, selected here because they are of similar size and exemplify genera inside and outside of hotnode clades, *Crotalaria* and *Copaifera*. *Crotalaria* is represented by 42 species in Brazil, five of which are used based on 15 use reports. Use of these species in Brazilian ethnomedicine is for very specific applications, including as abortifacients or vermifuges. *Crotalaria* species contain the pyrrolizidine alkaloid (PA) monocrotaline, which has been investigated as an anti-tumor agent (Kusuma et al., 2014). In contrast, the 26 species of *Copaifera* in Brazil, known to be of mild effect, have 70 use reports for six species. *Crotalaria*, unlike *Copaifera*, does not belong to a hotnode clade but is nested in a clade with *Sophora* (1 species, one use report), *Ulex* (1 species, not used), *Sellocharis* (1 species, not used) and *Lupinus* (31 species, none used), all plants with PAs (Wink, 2013). *Copaifera* and its close relatives in the hotnode clade (*Eperua, Hymenea*; **Figure 2**) contain diterpene resins with antimicrobial activity. These data exemplify the infrequent traditional use of toxic species and their relatives, compared to the species of mild effect in common use. If novel drug leads from the Leguminosae are developed for their cytotoxicity, then bioprospecting effort should be focused on species outside of hotnode clades. In the case of *Crotalaria*, ultimately monocrotaline was shown to be a hepatocarcinogen which causes pulmonary hypertension and veno-occlusive disease (e.g., Knutsen et al., 2017). Although many plants of high toxicity do not ultimately become commercialized therapeutics, the potential of more toxic products might explain the predominance of plants outside of hotnode clades in the FDA lists (**Table 2**). We find that although fewer ethnomedicinal species outside of hotnode clades are studied, but when they are there are more studies published. This finding might further support the view that these plants are toxic or have potential as leads.

Whether or not novel leads remain to be discovered, there are compelling reasons to characterize plants that represent different lineages in ethnomedicine. Lack of knowledge may be problematic because health risks associated with ethnomedicinal plant use may be unreported (Jäger, 2015). Studies on safety, and especially well-conducted clinical trials, are rare in Brazil (Dutra et al., 2016). Mazzari and Prieto (2014) highlighted little attention, in the Brazilian context, to the safety of herbal medicines. We show studies characterizing plants are most often of commonly used species from hotnode clades. For example, Brazilian species belonging to the Leguminosae genera *Stryphnodendron* (Costa et al., 2013), *Erythrina* (Dias et al., 2013), and *Pterodon* (Dutra et al., 2016), including plants used extensively in local medicine, have been the focus of recent pharmacological and toxicological survey without substantiating concerns. *Stryphnodendron adstringens* and *Erythrina mulungu* are listed by the Brazilian Health Ministry as priority species, and are found in our hotnode clades; *Caesalpinia ferrea* and *Bauhinia variegata* are also found in hotnode clades and are listed by the Brazilian Health Ministry. Plants outside of the hotnode clades are particularly poorly known, and it is notable that the only plant of family Leguminosae in the Brazilian Health Ministry list that is not either a common and global agricultural food or fodder plant or in a hot node is *Dalbergia subcymosa*, a plant with few reported uses in women’s medicine and relatively poorly known from a pharmacological or phytochemical perspective. Phylogenetic methods could be used in tandem with ethnobotanical databases to predict the health risks associated with uncharacterized plant species. Here we show that lineages rich in ethnomedicinal use have more ethnomedicinal species that are the focus of pharmacological studies, showing effort is directed towards characterizing closely-related, most-used species. Just as for bioprospecting purposes, it could be argued that phylogenetically-isolated species should be consciously included in future research. Plants outside of hotnode clades may raise more concerns for the safety of the users of traditional medicine, though they are less used.

Our study considered the presence or absence of ethnomedicinal use, and the presence or absence of phytochemical or pharmacological studies. We also considered intensity of ethnomedicinal use, where the number of use reports served as a proxy for intensity of use. Research effort was recorded by recording the number of published studies we found in our online searches. We are confident in our estimation of presence of ethnomedicinal use, since we make a thorough survey of literature and herbarium records (Souza and Hawkins, 2017). We consider number of use reports to be a reasonable estimate of ethnomedicinal intensity of use, though in the case of flowers, fruits, and seeds, their infrequent use may be their seasonal availability may reduce the number of reports (Benko-Iseppon and Crovella, 2010). Counting papers to estimate research effort may be more flawed. Firstly, it is difficult to make a complete search when publications might use a range of very broad or specific key words. Further, the actual studies may be more revealing than the fact that they exist. Although many pharmacological studies were found for the ethnomedicinal species, the quality and significance of the studies were not easily assessed. Many low impact studies use meaningless bioassays, and report differences between in vitro and in vivo experiments that are a consequence of data quality (Gertsch, 2009). Finally, we explore only ethnomedicinal use and phylogeny as potential factors contributing to the selection of a species for phytochemical or pharmacological studies. Other factors may be important, for example the availability of plentiful plant material (Atanasov et al., 2015); we note the species that are the most intensively screened often have food or fodder use, for example *Canavalia* species are used as food for cattle. Existing protocols and emerging interest may create a snowball effect, so that many studies are made of species of already proven interest, or of their close relatives.

Brazil is a megadiverse country with a rich history of plant use for medicine, but this knowledge and natural resource is yet to be transformed into products. Although ultimately the regulatory and research infrastructure together determine the success or failure of the drug development pipeline, here we consider aspects at the interface between traditional knowledge and biodiversity. This study shines a light on the phylogenetic distribution of ethnomedicinal plants in family Leguminosae from Brazil. In highlighting an overdispersed structure for the first time it draws attention to the complex nature of plant selection for ethnomedicinal use. It also highlights a connection between lineages of importance for ethnomedicine and those that have become the focus of ethnopharmacological research, raising questions about the drivers of selection of species for screening in pharmacological studies. Much has been written about means devising rational, data-driven selection of plants for screening, for example by inferring useful phytochemical composition from ethnobotanical, chemosystematic or ecological information (Coley et al., 2003; Tan et al., 2006; Douwes et al., 2008; de Albuquerque, 2010; Saslis-Lagoudakis et al., 2011; Albuquerque et al., 2012), or from phylogeny (Rønsted et al., 2008; Saslis-Lagoudakis et al., 2012; Grace et al., 2015; Yessoufou et al., 2015; Ernst et al., 2016). Ultimately, our study demonstrates a method to identify the plant lineages that are most important ethnomedicinally. As research into natural products grows, this framework provides novel insights into how knowledge of ethnomedicinal plants is distributed and might be used.

## Author Contributions

ENFS and JAH designed the study. All authors contributed to the analysis of the results and to the writing of the manuscript.

## Conflict of Interest Statement

The authors declare that the research was conducted in the absence of any commercial or financial relationships that could be construed as a potential conflict of interest.

## Footnotes

1 http://splink.cria.org.br/
