# Supplementary material for: Which Plants Used in Ethnomedicine Are Characterized? Phylogenetic Patterns in Traditional Use Related to Research Effort
Source: Front Plant Sci. 2018 Jun 20;9:834. doi: 10.3389/fpls.2018.00834 (PMC6019821; doi:10.3389/fpls.2018.00834)
Supplement: Supplementary file 2 [file Table_2.DOCX]

Supplementary Table 2. MPD and MNTD values for therapeutic applications, plant parts used and modes of application of the ethnomedicinal medicinal uses of the Leguminosae in Brazil. DCS - Diseases of the circulatory system, DDS - Diseases of the digestive system; DFS - Diseases of the femalegenito system; DGS - Diseases of the genitourinary system; DMC - Diseases of the musculoskeletal system and connective tissue; DRS - Diseases of the respiratory system; ENM - Endocrine, nutritional and metabolic diseases; IPD - Certain infectious and parasitic diseases; OTHER- not classified diseases such as fever, pain and inflammation. *ns=not significant.

|  |  |  | MPD |  |  | MNTD |  |
| --- | --- | --- | --- | --- | --- | --- | --- |
|  | ntaxa | mpd.obs | NRI | p-value | mntd.obs | NTI | p-value |
| DCS | 39 | 1.650 | -2.979 | <0.05 | 0.392 | -1.666 | ns |
| DDS | 75 | 1.590 | -2.295 | <0.05 | 0.206 | -0.269 | ns |
| DFS | 39 | 1.432 | 0.131 | ns | 0.314 | -0.535 | ns |
| DGS | 32 | 1.615 | -2.270 | <0.05 | 0.282 | 0.256 | ns |
| DMC | 42 | 1.625 | -2.634 | <0.05 | 0.275 | -0.183 | ns |
| DRS | 62 | 1.587 | -2.079 | <0.05 | 0.207 | 0.085 | ns |
| ENM | 38 | 1.429 | -0.421 | ns | 0.409 | -1.284 | ns |
| IPD | 37 | 1.576 | -1.879 | <0.05 | 0.340 | -0.821 | ns |
| OTHER | 108 | 1.584 | -2.226 | <0.05 | 0.165 | -0.042 | ns |
| Medicinal | 276 | 1.653 | -3.991 | <0.05 | 0.168 | -2.276 | <0.05 |
